# AI-HOPE: an AI-driven conversational agent for enhanced clinical and genomic data integration in precision medicine research

**DOI:** 10.1093/bioinformatics/btaf359

**Published:** 2025-06-19

**Authors:** Ei-Wen Yang, Enrique Velazquez-Villarreal

**Affiliations:** PolyAgent, San Francisco, CA 94102, United States; Department of Integrative Translational Sciences, Beckman Research Institute, City of Hope Comprehensive Cancer Center, Duarte, CA 91010, United States

## Abstract

**Motivation:**

The growing complexity of clinical cancer research has fueled a surge in demand for automated bioinformatics tools capable of integrating clinical and genomic data to accelerate discovery efforts.

**Results:**

We present the Artificial Intelligence Agent for High-Optimization and Precision Medicine (AI-HOPE), an AI-driven system that enables domain experts to conduct integrative data analyses through natural language interactions. Powered by Large Language Models, AI-HOPE interprets user instructions, converts them into executable code, and autonomously analyzes locally stored data. It supports flexible association studies, subset comparisons, clinical prevalence assessments and survival analyses. In addition, AI-HOPE enables global variable scans to identify features significantly associated with a user-defined outcome, making a powerful and intuitive tool for advancing precision medicine research. Importantly, its closed-system design prevents clinical data leakage. To demonstrate its utility, AI-HOPE was applied to The Cancer Genome Atlas data to address two clinical questions. First, it identified significant enrichment of TP53 mutations in late-stage colorectal cancer compared to early-stage cases. Second, it uncovered a strong association between KRAS mutations and poorer progression-free survival in FOLFOX-treated patients. These findings align with established literature and demonstrate AI-HOPE's ability to generate meaningful insights independently, without prior assumptions. By removing programming barriers and simplifying complex analyses, AI-HOPE bridges the gap between data complexity and research needs. With its scalable and adaptable framework, AI-HOPE has the potential to support diverse biomedical research fields, driving innovation and efficiency in translational studies.

**Availability and implementation:**

The AI-HOPE software and demonstration data is available at https://github.com/Velazquez-Villarreal-Lab/AI-HOPE.

## 1 Introduction

Precision medicine is revolutionizing healthcare by tailoring treatments to individual genetic, environmental, and lifestyle factors (Jameson and Longo 2015). At the core of this approach lies integrative analyses based on clinical and genomic data, providing a comprehensive understanding of disease mechanisms and therapeutic responses. However, the vast volume and complexity of such data present significant challenges for clinical research (Ashley 2015). Extracting meaningful insights requires advanced bioinformatic analyses to interpret genomic data alongside clinical metadata (e.g. age, gender, etc.) and treatment outcomes (e.g. survival data.) Unfortunately, standard bioinformatics analyses often require programming expertise, posing a barrier for many clinical researchers due to time constraints and technical demands.

To address these challenges, automated tools capable of end-to-end bioinformatics analyses are urgently needed to streamline clinical research workflows. Recent advances in Large Language Models (LLMs) have revolutionized artificial intelligence applications in biology, enabling transformative capabilities such as disease diagnosis and drug discovery (Flam-Shepherd *et al.* 2022). A key feature of LLMs is their conversational interface, which enables users to provide instructions in natural language to perform complex tasks. This approach surpasses traditional GUI-based platforms and offers fully automated solutions. (Zhou *et al.* 2024). Several LLM agent systems have emerged for omics data analysis. For example, CellAgent demonstrated efficacy in automating single-cell RNA sequencing data processing (Xiao *et al.* 2025), while tools like AutoBA offer conventional multi-omics analysis capabilities (Zhou *et al.* 2024).

Despite recent advances, the analytical needs of clinical research remain largely unmet. Current LLM-based tools lack the ability to seamlessly integrate clinical metadata with genomic information and treatment outcomes. State-of-the-art web-based platforms such as Xena (Goldman *et al.* 2020) and cBioPortal (Cerami *et al.* 2012) enable users to upload and analyze their own data but are constrained by predefined analytical modules and graphical user interfaces. These limitations hinder user-driven exploration and restrict the flexibility needed for critical clinical research applications, including biomarker discovery, disease progression modeling, and treatment efficacy evaluation (see Supplementary Results and Supplementary Table S1, available as supplementary data at *Bioinformatics* online for further comparison). To overcome these constraints, we introduce the Artificial Intelligence Agent for High-Optimization and Precision Medicine (AI-HOPE), an LLM-powered agent system that enables flexible association studies and case-control analyses via natural language queries. AI-HOPE offers a dynamic and customizable analytic environment designed to support the evolving demands of precision medicine research. AI-HOPE utilizes a locally deployed Llama3 model. This close-system setup avoids online data exchange and ensures compliance with Health Insurance Portability and Accountability Act (HIPAA) or General Data Protection Regulation (GDPR) protocols. The primary objective of AI-HOPE is to deliver essential statistical outputs, such as odds ratios, hazard ratios, and Kaplan-Meier survival curves, to advance discovery efforts in clinical research.

The capabilities of AI-HOPE were demonstrated through two analytical studies using data from The Cancer Genome Atlas (TCGA). The first study analyzed TP53 mutation enrichment in early-stage versus late-stage colorectal cancer (CRC) patients, where AI-HOPE identified a significant enrichment of TP53 mutations in late-stage (III/IV) CRC compared to early-stage (I/II) cases. The second study examined survival data for patients treated with the combination chemotherapy FOLFOX. Using AI-HOPE, we compared patients with and without KRAS mutations. The analysis revealed a significant association between KRAS mutations and poorer progression-free survival (PFS). The results highlight its capability to generate actionable insights that align with established scientific findings. Overall, AI-HOPE offers a scalable and intuitive framework for autonomous clinical data analysis. It is adaptable beyond cancer research and supports a wide range of applications in other biomedical fields. Future enhancements, such as including additional statistical tools and multi-omics capabilities, will further solidify its role as a pivotal resource for advancing clinical research and improving patient outcomes.

## 2 Methods

**Figure 1.**
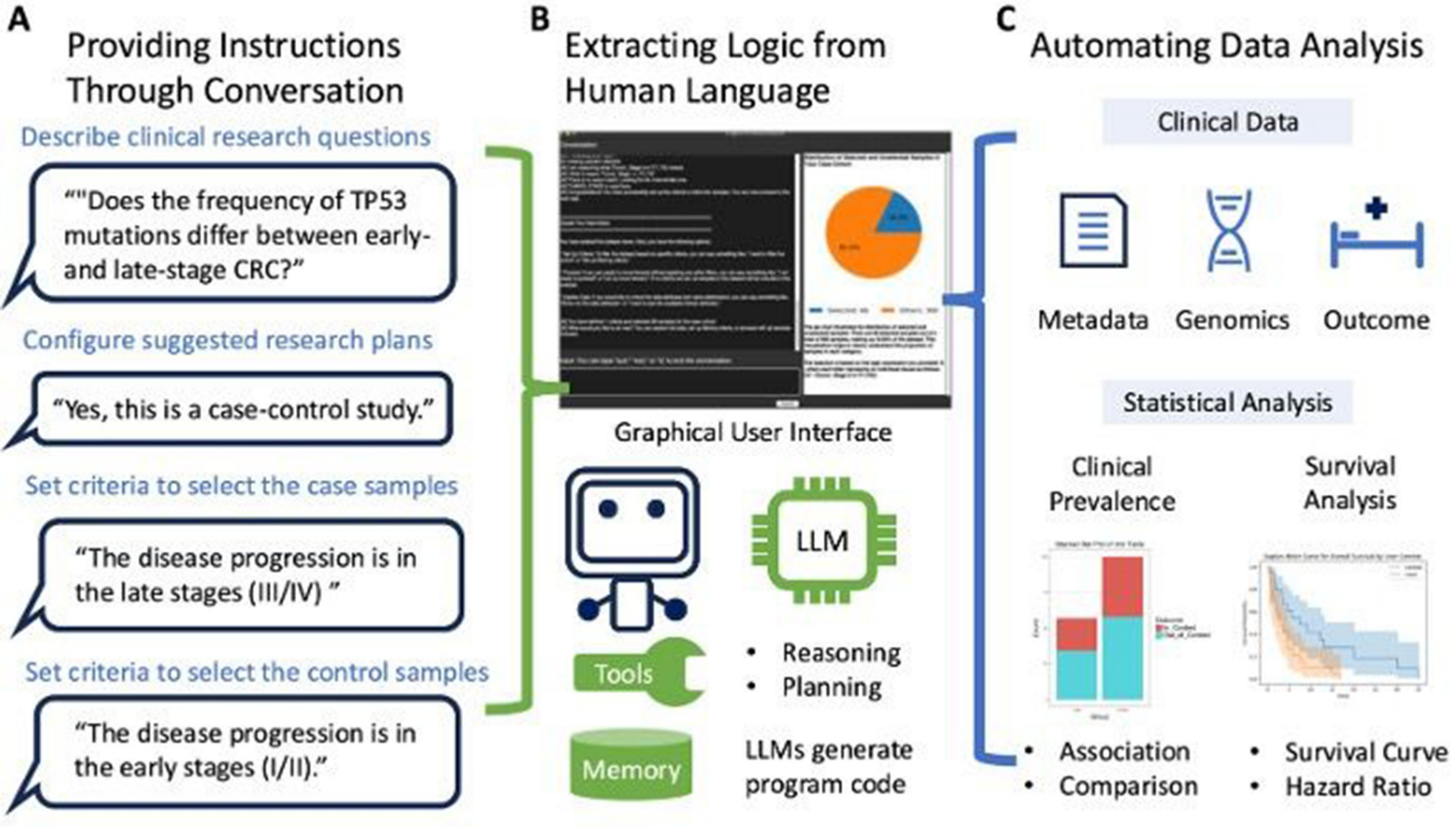
Overview of the Artificial Intelligence Agent for High-Optimization and Precision Medicine (AI-HOPE) Workflow. This figure illustrates the end-to-end workflow of the AI-HOPE system, which begins by interpreting users’ natural language questions and guiding them through optimized research strategies. (A) Conversational setup and configuration: users interact with AI-HOPE via a graphical user interface (GUI), submitting queries in natural language. The system assists in configuring the study design by recommending appropriate datasets, defining cohorts, and selecting statistical methods based on the user’s research objective. (B) Logic extraction and task translation: AI-HOPE uses LLM-powered agents to translate user intent into executable logic. It automatically generates code for data manipulation and applies reasoning and memory tools to ensure accurate and context-aware task execution. (C) Automated multi-modal data analysis: AI-HOPE conducts integrative analyses across clinical, genomic, and outcome data. It supports a range of analytical tasks, including prevalence testing, association analysis, and survival modeling. The system produces interpretable outputs such as odds ratios, *P*-values, survival curves, and hazard ratios—facilitating rapid, hypothesis-driven insights for precision medicine research.

AI-HOPE agent for precision medicine in clinical research: AI-HOPE is an AI-powered assistant that enables researchers to analyze clinical data using natural language input. Users simply describe their research question or task in a few sentences, and AI-HOPE automatically identifies the appropriate analysis type-either a data association analysis or a case-control study—and suggests a customized analysis plan than can be refined as needed. For example, a user might ask, “Do patients with KRAS mutations have different progression-free survival?” to initiate a case-control study, or “Is BRAF mutation linked to brain metastasis in melanoma?” for a data association analysis. AI-HOPE also supports automated discovery tasks such as, “Tell me everything associated with overall survival in colon cancer,” scanning all available variables to identify significant associations. By converting natural language into structured analytical workflows, AI-HOPE empowers users to explore data, uncover insights, and refine hypotheses—without needing to write a single line of code (Fig. 1, Supplementary Figs S1 and S2, available as supplementary data at *Bioinformatics* online).

Data preparation: to prepare input data for analysis, users can either manually organize their files or use the built-in utility app provided with the AI-HOPE software. Each dataset is placed into a designated folder that includes three key components: a README file, an index file, and a main tab-delimited data table. The README provides an overview of the dataset, the index file lists key attributes available for analysis, and the data table contains the core data, with samples as rows and attributes as columns. To demonstrate the potential of AI-HOPE agents in advancing precision medicine in oncology, we integrated data from The Cancer Genome Atlas (TCGA) via UCSC Xena (Goldman *et al.* 2020) and cBioPortal (Cerami *et al.* 2012). However, AI-HOPE is not limited to TCGA data—users can easily upload and analyze their own datasets, provided they follow the required input format. The platform is designed with flexibility in mind and supports a wide range of clinical and genomic data types. All example datasets used in this study are available for download via the AI-HOPE SharePoint.

Load and subset data through natural language queries: the AI-HOPE workflow typically begins by prompting users to specify the dataset name, followed by an optional sample selection step that allows for subsetting the data or defining case and control groups. Users can define sample selection criteria using natural language statements, each of which is parsed into a logical expression composed of three elements: a data attribute, a comparison operator, and a value. For example, in the statement “Age is greater than 30,” Age is the attribute, is greater than is the comparison operator, and 30 is the value. AI-HOPE supports a wide range of comparison operators, including equality (e.g. “is,” “is not”), inequality (e.g. “greater than,” “less than”), ranges (e.g. “from [Start Value] to [End Value]”), and set inclusion/exclusion (e.g. “is in,” “is not in”). For instance, the expression “Disease stage is in {Stage I, Stage II, Stage III}” defines a valid inclusion criterion for multiple categorical values. By following these structured rules, AI-HOPE accurately converts natural language statements into executable arithmetic expressions, ensuring reliability in sample selection. For a detailed evaluation of this process, refer to the Supplementary Methods and Supplementary Table S4, available as supplementary data at *Bioinformatics* online. Note that incomplete or redundant user inputs may increase the risk of hallucinations by the LLM. Recommended strategies for managing these scenarios are detailed in the Supplementary Results and summarized in Supplementary Table S5, available as supplementary data at *Bioinformatics* online. When multiple statements are included, each statement should be enclosed in parentheses for clarity. Statements can be joined using logical operators “and” and “or,” allowing the construction of complex expressions. For example, “(Age is greater than 30) and (Gender is male) or (ETHNICITY is Hispanic)” combines multiple statements. AI-HOPE evaluates the statements based on their positions, from left to right. Nested parentheses can be used to override the default evaluation order to ensure specific priorities. This intuitive approach simplifies data stratification, enabling researchers to interact with the data using natural language.

Data lookup and autonomous association analysis with AI-HOPE: AI-HOPE supports both data exploration and automated association analyses through natural language input. Users can perform data lookups to visualize the distribution of numerical and categorical variables, as illustrated in Supplementary Fig. S3, available as supplementary data at *Bioinformatics* online. Beyond basic querying, AI-HOPE enables statistical association studies between any pair of clinical variables, as well as between clinical features and survival outcomes. The system autonomously selects the most appropriate statistical test based on the variable types involved (see Supplementary Methods for details, available as supplementary data at *Bioinformatics* online). AI-HOPE also supports global association scans, allowing users to identify variables significantly associated with a given target, including survival endpoints. For instance, when prompted with “Find all variables associated with KRAS mutation status in the TCGA COAD dataset,” AI-HOPE identified significant associations with MSI status, BRAF mutation, and multiple copy number variation features (Supplementary Fig. S4, available as supplementary data at *Bioinformatics* online). To explore further, the follow-up query “Correlate BRAF mutation status with KRAS mutation in colon cancer” revealed a mutually exclusive relationship between the two mutations—consistent with previously published findings in colorectal cancer.

Flexible Case-Control Grouping and Context-Aware Comparisons in AI-HOPE: Another major strength of AI-HOPE is its flexibility in defining case and control groups based on user-specified clinical criteria, such as mutation status, tumor stage, or demographic characteristics. Once the groups are defined, AI-HOPE enables comparisons across a broad spectrum of variables—including genomic, clinical, and outcome measures such as survival. Users can also define custom analytical contexts by combining multiple conditions. AI-HOPE evaluates these context-aware groupings by comparing the frequency of in-context versus out-of-context samples across the case and control cohorts. For example, researchers can assess whether Hispanic male patients—identified by combining ethnicity and gender attributes—are overrepresented in the responder group compared to the non-responder group using an Odds Ratio Test. In addition to user-defined comparisons, AI-HOPE can perform automated scans across all available variables to identify those significantly associated with the defined case-control structure. This functionality streamlines exploratory analysis and supports hypothesis generation for precision oncology research.

## 3 Results

### 3.1 Applications of precision medicine in cancer

#### 3.1.1 The enrichment of TP53 mutations in early-stage versus late-stage colorectal cancer

In our first use case, we promped AI-HOPE with the question: “Compare TP53 mutations in early-stage versus late-stage colorectal cancer.” The agent recommended conducting a case-control study, comparing TP53 mutation frequency between early-stage (Stage I/II) and late-stage (Stage III/IV) CRC patients. To define the case group (late-stage), we applied the criterion: “TUMOR_STAGE is in {T3, T4, T4A, T4B},” where TUMOR_STAGE refers to the AJCC pathological tumor stage. For the control group (early-stage), the criterion was “TUMOR_STAGE is in {T1, T2}.” Following AI-HOPE's guidance, we tested whether samples with “TP53_Mutation_status = 1”—indicating a mutation in the TP53 gene—were significantly enriched in the late-stage cohort using an odds ratio test. AI-HOPE identified that TP53 was mutated in 65.77% of late-stage patients compared to 50.0% of early-stage patients, with a statistically significant *P*-value of .024. This finding indicates a significant enrichment of TP53 mutations in late-stage CRC and aligns with previously published studies, including findings by Iacopetta *et al.* (2006), thereby supporting the analytical accuracy and validity of AI-HOPE (Supplementary Fig. S5, available as supplementary data at *Bioinformatics* online).

#### 3.1.2 Survival outcomes among FOLFOX-treated patients with or without KRAS mutations

In another demonstration, we ask AI-HOPE: “Compare survival outcomes between FOLFOX-treated patients with and without KRAS mutations in colon cancer.” The FOLFOX regimen consists of fluorouracil (5-FU), leucovorin calcium (folinic acid), and oxaliplatin. To define the case cohort, we applied the following criteria: (Agent is in {Fluorouracil, Leucovorin, Oxaliplatin}) and (KRAS_mutation_status is 1)—indicating patients who received FOLFOX and had a KRAS mutation. The control cohort was defined as: (Agent is in {Fluorouracil, Leucovorin, Oxaliplatin}) and (KRAS_mutation_status is 0)—patients who received FOLFOX but did not carry a KRAS mutation. The variable Agent represents the treatment components, while KRAS_mutation_status denotes mutation presence (1) or absence 0 in the KRAS gene. AI-HOPE’s analysis revealed that patients with KRAS mutations had significantly worse survival outcomes compared to those without the mutation, indicating a poorer response to FOLFOX and a higher risk of recurrence (Supplementary Fig. S6, available as supplementary data at *Bioinformatics* online). These findings are consistent with prior literature, including observations by Di Nicolantonio *et al.* (2021), demonstrating AI-HOPE’s capability to independently generate clinically meaningful insights.

## 4 Conclusions

AI-HOPE streamlines precision medicine research by integrating clinical and genomic data through a user-friendly, natural language interface powered by LLMs. It enables advanced analyses without the need for programming expertise and has already demonstrated success in applications such as evaluating TP53 mutations in CRC and analyzing KRAS-related treatment outcomes. AI-HOPE runs locally, ensuring that sensitive clinical data remains secure and protected from potential exposure through online data exchange. Its adaptable architecture supports a wide range of use cases, including biomarker discovery, disease mechanism research, and therapeutic response evaluation across diverse disease types. While platforms like Xena and cBioPortal support full multi-omics integration, AI-HOPE currently focuses on linking genomic and clinical data. However, the system is designed with extensibility in mind and can be expanded to incorporate additional omics layers, such as transcriptomics and proteomics. Future development plans also include the implementation of missing data handling and integrated quality control features. Additionally, the creation of a web-based version is underway to further enhance accessibility and usability across research settings.

## Supplementary Material

btaf359_Supplementary_Data
