# Supplementary material for: AI-HOPE: an AI-driven conversational agent for enhanced clinical and genomic data integration in precision medicine research
Source: Bioinformatics. 2025 Jun 19;41(7):btaf359. doi: 10.1093/bioinformatics/btaf359 (PMC12212640; doi:10.1093/bioinformatics/btaf359)
Supplement: btaf359_Supplementary_Data [file btaf359_supplementary_data.docx]

**AI-driven conversational agent enhances clinical and genomic data integration for precision medicine research**

**Ei-Wen Yang, Ph.D.^1^, Enrique Velazquez-Villarreal, M.D., Ph.D., M.P.H.^2,3,^***

^1^PolyAgent, San Francisco, CA

^2^Department of Integrative Translational Sciences, Beckman Research Institute of City of Hope, Duarte, CA

^3^City of Hope Comprehensive Cancer Center, Duarte, CA

*Corresponding Author. Email: evelazquezvilla@coh.org

**Supplementary Methods**

Architecture of the AI-HOPE Agent Framework

**AI-HOPE functions as an intermediate software layer that bridges user input with complex bioinformatics analysis workflows by leveraging large language models (LLMs). The system is built on a top-down hierarchical architecture, centered around a supervisor agent that coordinates task execution and information flow.** As illustrated in Figure S2, the supervisor agent uses LangGraph to manage interactions among key system components—including the user interface, LLM, memory modules, analytical tools, and specialized data analysis agents. AI-HOPE operates using the pretrained Meta-Llama-3-8B-Instruct model, without additional fine-tuning. Instead, it relies on prompt engineering and a structured workflow to guide the LLM’s behavior and ensure consistent task execution. The agent framework includes two core analytical agents. The group analysis agent assists in defining comparison groups, assessing differences across data attributes, and identifying clinical variables associated with those groupings. The data exploration agent enables intuitive visualization of data distributions and associations, supporting hypothesis generation and validation within the dataset. Together, these agents form a cohesive and extensible framework for conversational, AI-powered precision medicine research.

Statistical Framework for Association Analysis in AI-HOPE

AI-HOPE evaluates associations between a clinical variable of interest and other attributes using statistical methods tailored to the data types involved. For associations between two categorical variables, the system applies the Chi-square test. When comparing a categorical variable with a numerical variable, AI-HOPE uses the Mann-Whitney U test for two-group comparisons and the Kruskal-Wallis test for comparisons involving more than two groups. Associations between two continuous variables are assessed using Spearman’s rank correlation, with meaningful relationships defined by Spearman’s rho > 0.3 and p-value < 0.05. For survival outcomes, AI-HOPE employs Cox proportional hazards models to evaluate the prognostic significance of each clinical attribute in relation to overall survival (OS) and progression-free survival (PFS). Statistical significance is determined using p-values, and false discovery rate (FDR) correction is applied to account for multiple comparisons. These analytical approaches follow established best practices commonly used in large-scale studies, including those conducted by The Cancer Genome Atlas (TCGA). Attributes with p-values < 0.05 are considered potentially significant and are flagged for further investigation. Results are presented in a structured output table, where each row corresponds to a numerical variable or categorical level. Each entry includes a summary of the statistical findings and an indicator denoting whether the model successfully executed the analysis.

Software Availability and User Accessibility

To enhance usability and facilitate widespread adoption, we developed one-command auto-installers compatible with both Mac and Windows operating systems. Additionally, the software package includes a utility script that enables users to upload their own datasets in spreadsheet format directly into the AI-HOPE environment. This script performs automatic format validation to minimize preprocessing requirements and ensure smooth integration. In compliance with the editor’s request and to promote transparency and reproducibility, the full AI-HOPE software package has been made publicly available via Zenodo and can be accessed at the following link: https://zenodo.org/records/15200392?token=eyJhbGciOiJIUzUxMiJ9.eyJpZCI6IjFlZjllYzc1LTkwOWUtNDJlNS1iZmFmLTc0Y2FhZjg2ZjU2NyIsImRhdGEiOnt9LCJyYW5kb20iOiIzNzYyYTk1ZDFhYTU5NjZkY2U4NzNlMTExYTlmYzBjZiJ9.UjQubOFtPxAmJ-_fs74ei5lh6bXQbb5cV7yqfHBsLyVNw1SQYdPutjfsuDCF9_1GNVgu99TJwxKzeyBhbKnFlA.

Simulated Data for Performance Evaluation

The accuracy of AI-HOPE relies heavily on its ability to convert natural language statements into valid arithmetic expressions for sample subsetting. This involves correctly identifying three components from each input: the data attribute, comparator, and the value. To evaluate performance across varying input complexities, we simulated natural language queries using data from the TCGA colorectal cancer (CRC) dataset.

For positive test cases, each clause was constructed by randomly selecting a valid data attribute, a supported comparator (such as "is less than" or "is in"), and a corresponding value sampled from the dataset. These elements were then combined into valid natural language statements—for example, "Age is less than 50." Such inputs were labeled as positive (P), and AI-HOPE was expected to correctly identify and extract each clause's attribute, comparator, and value. A test case was considered successfully processed only if all clauses were interpreted correctly.

Negative instances were introduced to challenge the system’s ability to detect and reject invalid inputs. These were evenly divided into two main types. The first type, labeled N1, consisted of unrelated sentences that do not form logical expressions. These were randomly selected from a TCGA CRC publication [The Cancer Genome Atlas Network, et al. 2012] to ensure contextual relevance but semantic invalidity. The second type included logical expressions containing structural errors. This group was further divided into two subcategories: N2, which contained clauses missing one of the essential elements (attribute, comparator, or value), and N3, which included clauses with redundant or extraneous elements. These instances were designed to be true negatives, and AI-HOPE was expected to reject them without attempting to extract components.

This simulation framework provided a rigorous means of evaluating AI-HOPE’s natural language understanding, logical parsing capabilities, and robustness across varying levels of input complexity.

**Supplementary Results**

AI-HOPE Supports Flexible Experimental Design for Advanced Clinical Research

While cBioPortal and Xena are widely recognized as state-of-the-art platforms for integrative analysis of clinical, genomic, and treatment outcome data, both have limitations when it comes to flexible, user-defined experimental design. AI-HOPE addresses this gap by offering a conversational interface that supports a broader range of analytical configurations tailored to user-defined research questions. For example, AI-HOPE enables global association studies in which users can search for all variables associated with a selected attribute of interest, such as tumor mutational burden (TMB) or microsatellite instability (MSI) status. In contrast, cBioPortal restricts association analyses to pre-defined sample groups, and Xena lacks support for systematic variable-by-variable association scans altogether.

Additionally, AI-HOPE supports context-aware comparisons by allowing users to define clinical subgroups using multiple criteria—such as combining ethnicity and gender to isolate Hispanic male patients—and compare their distribution across different clinical outcomes (e.g., responders vs. non-responders). While Xena offers limited support for such stratified comparisons, cBioPortal does not provide this functionality. This level of customization is critical for precision medicine, where nuanced subgroup analyses can reveal factors influencing treatment efficacy and disease progression. Overall, AI-HOPE offers greater flexibility and supports more complex, multi-variable experimental designs than either Xena or cBioPortal. Its LLM-powered conversational interface enables researchers to construct sophisticated workflows with minimal technical overhead. A detailed comparison of these platforms' experimental design capabilities is presented in Supplementary Table S1A.

AI-HOPE Enables Advanced Survival Analysis for Clinical Research

Survival analysis is a cornerstone of clinical research, playing a critical role identifying therapeutic targets, uncovering prognostic biomarkers, and evaluating treatment outcomes. While AI-HOPE, Xena, and cBioPortal all support basic Kaplan–Meier (KM) survival plots, only AI-HOPE and cBioPortal provide functionality for Cox proportional hazards regression to calculate hazard ratios. What sets AI-HOPE apart is its ability to perform automated survival analysis across all available clinical variables—capability not supported by either Xena or cBioPortal due to their more rigid experimental design frameworks. Furthermore, AI-HOPE supports multivariable Cox regression, allowing users to adjust for covariates and generate more accurate, clinically meaningful interpretations. For visualization, AI-HOPE enhances result accessibility through the generation of forest plots, which clearly summarize survival associations across multiple variables. These advanced analytical and visualization features make AI-HOPE a more comprehensive and user-friendly tool for conducting survival analysis in translational research settings. A detailed comparison of survival analysis features across AI-HOPE, Xena, and cBioPortal is provided in Supplementary Table S1B.

Autonomous Statistical Results from AI-HOPE Perfectly Align with State-of-the-Art Web-Based Tools

AI-HOPE, Xena, and cBioPortal are widely used tools for analysing clinical datasets, designed to report statistical significant associations rather than to perform predictive modeling. Their primary function is to quantify relationships between variables using standard statistical tests, with results reported as p-values. Xena and cBioPortal have been extensively validated and are considered benchmarks in the biomedical research community. As such, comparing the statistical outputs of AI-HOPE with those from these platforms under matched experimental conditions provides a robust means of assessing its analytical reliability.

To perform this evaluation, we applied AI-HOPE to clinical data from the TCGA COAD cohort using datasets from both UCSC Xena (e.g., GDC TCGA COAD) and cBioPortal (e.g., TCGA COAD PanCancer Atlas). It is important to note that these platforms differ slightly in data annotation. For example, Xena includes pre-annotated values for microsatellite instability (MSI) and tumor mutational burden (TMB), while cBioPortal provides only raw MSI and TMB scores. Accordingly, for analyses based on cBioPortal data, we stratified MSI and TMB scores by early- and late-onset status to align with the annotation structure used in AI-HOPE. The exact natural language queries used in AI-HOPE for this benchmarking exercise are listed in Supplementary Table S2. As shown in Supplementary Table S3, the p-values produced by AI-HOPE matched those from both Xena and cBioPortal across all comparisons. This result demonstrates the accuracy and consistency of AI-HOPE’s autonomous statistical engine and highlights its use of standard statistical libraries—specifically, the R surv package in Xena and the Python lifelines package in AI-HOPE—which implement equivalent survival analysis methods. These findings confirm that AI-HOPE can deliver results on par with well-established web-based tools while offering the added benefit of a flexible, locally deployable, conversational interface.

Performance Assessment Through Simulated Data Testing

To evaluate the performance of AI-HOPE across varying levels of input complexity, we generated 100 positive and 100 negative samples for each complexity level, defined by the number of logical clauses ranging from one to five. These simulated inputs, as previously described, were used to systematically assess the model’s ability to accurately interpret and process natural language queries under increasing analytical complexity. To minimize bias introduced by random sampling, the evaluation process was repeated ten times. For each input sentence, AI-HOPE either correctly identified the data attribute, comparator, and value, or rejected the input if it did not represent a valid logical expression.

Predictions were classified into four categories. A true positive (TP) occurred when AI-HOPE successfully extracted all required components from a valid input. A false positive (FP) was recorded when AI-HOPE incorrectly extracted components from an invalid input. If AI-HOPE failed to extract components from a valid input, it was labeled as a false negative (FN). Conversely, if AI-HOPE correctly rejected an invalid input, it was considered a true negative (TN). Based on these classifications, we calculated standard performance metrics to evaluate the system’s effectiveness. Accuracy was defined as 100% × (TP + TN) / (TP + TN + FP + FN). Precision was calculated as TP / (TP + FP), and recall as TP / (TP + FN). The F1 score was computed as the harmonic mean of precision and recall, using the formula: 2 × (Precision × Recall) / (Precision + Recall). These metrics provided a comprehensive assessment of AI-HOPE’s ability to accurately interpret natural language queries across varying levels of complexity..

$$F_{1}=2\cdot\frac{\text{Precision}\cdot\text{Recall}}{\text{Precision}+\text{Recall}}$$

The results are summarized in Table S4. Across all levels of input complexity, we observed no significant differences in overall accuracy. Remarkably, both the true positive accuracy and recall values remained at 100% across all 10 simulation runs. This demonstrates that when user inputs adhere to the expected format—providing valid attributes, comparators, and values—AI-HOPE reliably and consistently converts natural language into accurate arithmetic expressions. However, parsing time increased notably with input complexity. As the number of logical clauses grew, the average response time ranged from 2.35 to 11.91 seconds, reflecting the additional computational effort required by the LLM to process more complex queries.

Running Time Evaluation of AI-HOPE Across Task Types, Input Complexity, and Dataset Size

To assess the running time of AI-HOPE, we measured performance across different analytical task types, varying input complexities, and dataset sizes. All tests were conducted on a standard MacBook with an M2 chip, 16GB of unified memory, an 8-core CPU with 4 performance cores and 4 efficiency cores, and a 10-core GPU. Execution time s were recorded using datasets ranging from 50 to 5,000 samples, randomly drawn from the TCGA colorectal cancer (CRC) dataset. Two types of analyses were evaluated. The first, case-control analysis, involves parsing a natural language query to define case and control groups, followed by statistical comparison of a selected clinical variable between those groups. Simulated inputs containing one to five clauses were used to represent increasing levels of complexity, with a clinical variable randomly selected for each run. The second task type, data exploration analysis, does not require subsetting. Instead, a variable is selected at random, and its distribution is visualized across the full dataset or within the predefined groups. In total, six analysis settings were tested: five corresponding to group analyses with increasing input complexity, and one representing a data exploration task. Each setting was repeated ten times across all dataset sizes. Results are presented in Figure S6.

The findings show that increasing the dataset size from 50 to 5,000 samples resulted in only a modest increase in average execution time—approximately half a second—indicating that dataset size had minimal impact on overall performance within this range. The primary factor influencing execution time was input complexity; specifically, queries with multiple logical clauses required significantly more processing time in the group analysis settings.

Handling Imperfect User Inputs and Reducing Hallucinations in AI-HOPE

Hallucination is a known limitation of LLMs, including the LLaMA 3 model implemented in AI-HOPE. When hallucinations occur, the model may generate plausible but incorrect outputs rather than flagging an error. In our simulation study—designed to reflect typical real-world user behavior—our prompt templates achieved 100% accuracy when users submitted well-structured logical clauses containing valid attribute names, comparators, and values. However, hallucinations were more likely to occur when inputs included malformed clauses that mimicked valid syntax but contained missing or duplicated components, such as attribute names, operators, or values. N2 and N3 represent extreme examples of such cases, with hallucinations observed in an average of 51.4% of N2 and 76.9% of N3 queries. To mitigate these errors, we implemented a post-processing pipeline that verifies the existence of attribute names, checks the validity of comparators and values, and ensures that the resulting logic does not yield an empty sample selection. A comparative analysis of outcomes before and after applying these safeguards demonstrated substantial improvements in robustness—boosting accuracy by an average of 17.8% in N2 and 19.8% in N3 scenarios, as reported in Table S5. Nonetheless, residual errors may still occur. We therefore recommend that users review all AI-HOPE-generated analytical reports to confirm that input logic clauses are complete, accurate, and aligned with their intended analytical goals.

**References:**

1. The Cancer Genome Atlas Network. (2012) Comprehensive molecular characterization of human colon and rectal cancer. *Nature,* **487**, 330–337.

**Supplementary Tables**

**Table S1: Comparison of Experimental Design and Analysis Capabilities Across AI-HOPE, Xena, and cBioPortal.**

This table provides a side-by-side comparison of key features supported by AI-HOPE, Xena, and cBioPortal, with a particular focus on experimental design flexibility and survival analysis functionality. Table S1A evaluates each platform’s ability to support various types of experimental designs, including user-defined grouping and context-aware comparisons based on multiple clinical attributes. Table S1B highlights survival analysis capabilities, including support for Cox proportional hazards modeling, multivariable regression, hazard ratio reporting, and visualization features such as forest plots. Together, these comparisons underscore AI-HOPE’s enhanced flexibility and analytical depth in precision medicine research.

**Table S2. Clinical Queries Used for Evaluate AI-HOPE Performance.**

This table presents the six natural language input queries used to assess AI-HOPE’s performance, applied to clinical data from the TCGA COAD cohort. The queries focus on comparing microsatellite instability (MSI) status, tumor mutational burden (TMB), and their associations with survival outcomes between early-onset and late-onset colorectal cancer patients. These examples demonstrate AI-HOPE’s ability to interpret complex clinical questions and execute corresponding analytical workflows.

**Table S3. Comparison of P-Values Across AI-HOPE, Xena, and cBioPortal.**
This table presents the p-values generated by AI-HOPE, Xena, and cBioPortal for the six clinical queries outlined in Table S2. The results show complete concordance across all platforms, demonstrating that AI-HOPE delivers statistically identical outputs to those of the established tools. This consistency validates the reliability and analytical accuracy of AI-HOPE in clinical data analysis.

**Table S4: Performance metrics of AI-HOPE for Logical Expression Conversion**

(A) This section summarizes the overall performance of AI-HOPE in converting natural language statements into logical expressions, reporting precision, recall, and F1 scores. Standard deviations for each metric are shown in parentheses to reflect variability across simulation runs. (B) This section presents category-specific accuracy metrics for AI-HOPE based on simulated data, highlighting the model's ability to distinguish between true positives, false positives, true negatives, and false negatives. Standard deviations are provided in parentheses to indicate consistency across repeated evaluations.

**Table S5. Accuracy of AI-HOPE before and after post-processing in simulated data.**

This table presents the accuracy improvements resulting from the implementation of post-processing safeguards in the N2 and N3 categories. Results are further broken down into six error subtypes: Missing Attributes, Missing Comparators, Missing Values, Duplicated Attributes, Duplicated Comparators, and Duplicated Values. Standard deviations are shown in parentheses to reflect consistency across repeated evaluations.

**Supplementary Figures**

**Figure S1. User Interface of AI-HOPE.**

The interface is organized into three main sections: (A) Chat history, which logs the conversation between the user and the AI agents; (B) User input panel, where users submit analysis requests using natural language; and (C) Real-time analytics display, which presents the results of data analyses in the form of interactive reports.

**Figure S2. System architecture of the AI-HOPE agent framework.**
This figure illustrates the hierarchical architecture of AI-HOPE, centered around a supervisor agent that orchestrates the system's operations. The supervisor leverages LangGraph to manage communication between the user interface, the pretrained Meta-Llama-3-8B-Instruct model, memory components, external tools, and a suite of specialized data analysis agents. These agents include modules for group analysis and data exploration. AI-HOPE operates using prompt engineering and a structured workflow engine, without fine-tuning the base LLM, ensuring modularity, transparency, and adaptability across analytical tasks.

**Figure S3. Visualizing the distribution of numeric and non-numeric data columns**

(A) A histogram with an overlaid density curve illustrates the distribution of the numeric variable Age, showing the frequency and spread of values within the dataset. The x-axis represents age values, while the y-axis indicates their frequency. The smooth density curve highlights the central tendency and overall distribution shape. (B) Bar plots display the distribution of the categorical variable TMB status. The top panel shows the absolute counts for each category, while the bottom panel presents the corresponding percentage representation. The y-axis lists the category values (Elevated and Normal), and the x-axis reflects either the count or percentage, depending on the panel.

**
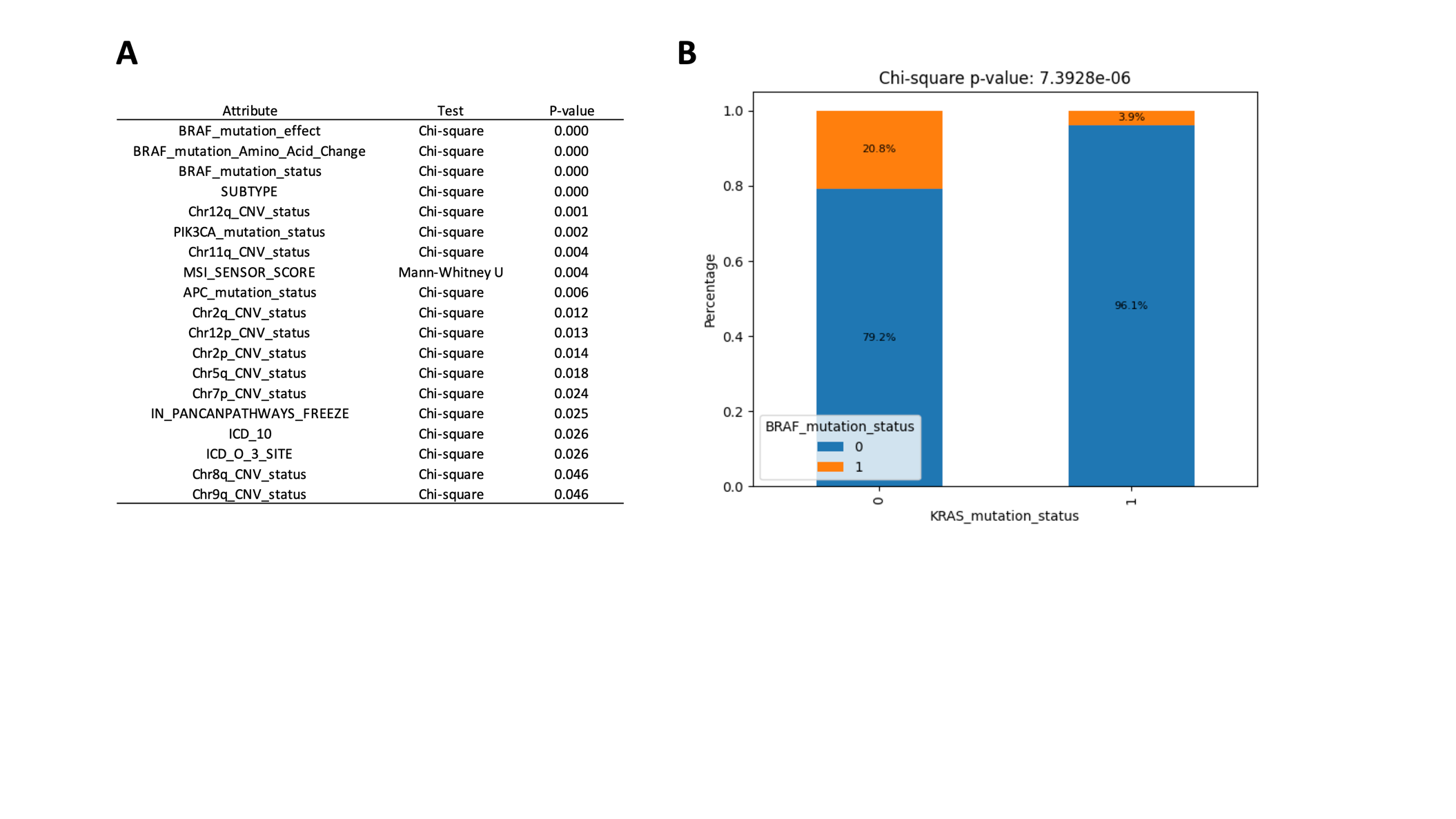
**

**Figure S4. Associations between KRAS mutations and molecular features in colorectal cancer**

**(A) A summary table listing all attributes found to be significantly associated with KRAS mutation status. Each row includes the attribute name, the statistical test used (e.g., Chi-square, Mann–Whitney U, or log-rank test), and the corresponding p-value, providing a clear overview of statistically significant associations. (B) A stacked bar plot illustrating the proportion of patients with BRAF mutations (shown in orange) across the KRAS-mutant and KRAS wild-type groups. The visualization highlights the well-established mutually exclusive relationship between KRAS and BRAF mutations in colorectal cancer, consistent with findings in the literature.**

**
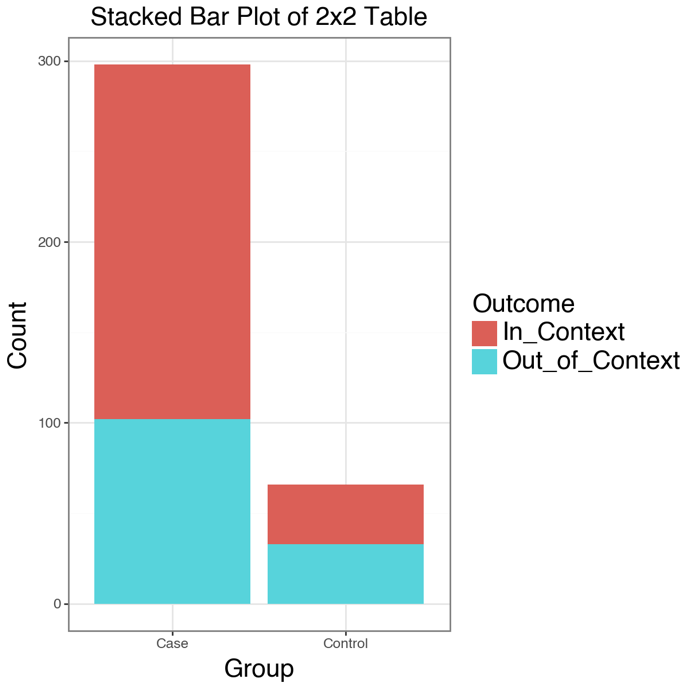
**

**Figure S5. Odds ratio analysis of TP53 mutation status in case and control groups**

Using the user-defined condition “TP53_Mutation_status is 1”, PM-AI conducted an odds ratio analysis to compare the frequency of TP53 mutations between case and control groups. A 2×2 contingency table was generated, and statistical significance was assessed using the Chi-square test. The results are visualized in a stacked bar plot and summarized in the accompanying PDF report. In total, 196 patients (65.77%) in the case group and 33 patients (50.0%) in the control group were classified as in-context samples. The Chi-square test yielded a p-value of 0.0237. The calculated odds ratio was 1.9216, with a 95% confidence interval of [1.12, 3.29], indicating a statistically significant enrichment of TP53 mutations in the case group.

**Figure S6. Kaplan-Meier Plots for survival analysis stratified by user-defined context**

(A) Kaplan–Meier plot comparing overall survival (OS) probabilities in FOLFOX-treated colorectal cancer (CRC) patients with and without KRAS mutations. (B) Kaplan–Meier plot comparing progression-free survival (PFS) probabilities for the same patient groups. (C) Forest plot displaying hazard ratios (HRs) for OS based on KRAS mutation status. (D) Forest plot displaying HRs for PFS based on KRAS mutation status.

In both Kaplan–Meier plots, the x-axis represents time in months, and the y-axis indicates survival probability. Shaded areas denote the 95% confidence intervals (CIs), reflecting the range within which the true survival estimates are likely to fall. Statistical significance between survival curves was assessed using the log-rank test, with p-values < 0.05 indicating a significant difference in survival outcomes based on KRAS mutation status. The forest plots summarize hazard ratios for both OS and PFS. Values greater than 1 indicate an increased risk of death or disease progression in the KRAS-mutant group compared to the wild-type group, while values less than 1 indicate a decreased risk. Error bars represent 95% confidence intervals for the HR estimates, providing a measure of the precision of these estimates. These analyses demonstrate the prognostic impact of KRAS mutation status in the context of FOLFOX treatment.

**Figure S7. Running time of AI-HOPE under different analysis settings and dataset sizes.**This figure illustrates the running time of AI-HOPE under varying analytical settings and dataset sizes. The x-axis represents the number of samples, ranging from 50 to 5,000, while the y-axis indicates execution time in seconds. Six analysis configurations are displayed. Case_Control_1 through Case_Control_5 correspond to increasing levels of input complexity in group analysis tasks, defined by the number of logical clauses in the user query. Data_Explore represents a data exploration task, which involves visualizing the distribution of a selected variable without subgroup comparison. Each data point reflects the average execution time across 10 repeated runs, providing a reliable measure of performance consistency. The results demonstrate how input complexity, rather than dataset size, is the primary factor influencing execution time.
